# Fluid-particle-structure interaction in single shot peening

**DOI:** 10.1038/s41598-024-63872-5

**Published:** 2024-06-06

**Authors:** Yusuke Mizuno, Takashi Misaka, Yoshiyuki Furukawa

**Affiliations:** Industrial Cyber-Physical Systems Research Center, National Institute of Advanced Industrial Science and Technology (AIST), 2-3-26 Aomi, Koto-Ku, Tokyo, 135-0064 Japan

**Keywords:** Fluid dynamics, Computational science

## Abstract

Shot peening is a widely used cold-working process. Physical phenomena of shot peening are analyzed using the developed fluid-particle-structure coupled solver. The influences of the flow field and shot peening parameters such as the shot impact velocity and shot size are investigated in the case of the falling, impacting, and rebounding single particle. The weakly coupled solver applies the immersed boundary method which enables direct evaluation of the interactions between the unsteady flow field and moving/deforming objects. The elastoplastic object of AISI4340 during the collision of rigid steel shot is analyzed dynamically using the finite element method. Consequently, it is clarified that the flow field of the post-collision between the shot and structure can be characterized by the relative Reynolds number, which is based on the shot diameter and relative velocity between the uniform flow and rebounding shot velocities. As the relative Reynolds number increases, the complex flow field and vortex structures are generated at the collision location. These fluid structures affect the collision phenomena resulting in the random behavior of the shot and the asymmetric indentation in the structure.

## Introduction

Shot peening is a cold-working technique used for the surface strengthening of metallic workpiece. During this process, multiple hard particles along with a jet stream from the nozzle collide with the surface of the workpiece. Consequently, compressive residual stress and work hardening are generated at the collision location of the workpiece. These effects are expected to prevent the fatigue fracture of the workpiece surface and provide wear resistance; therefore, shot peening is widely used as a finishing process. Particles, called shots, are made of metallic materials, such as steel. Depending on the materials and finishing conditions of the workpiece, the shot diameter and velocity are usually in the range of 0.2 to 1 mm and from 20 to 100 m/s, respectively. The workpieces are made of various metal materials such as aluminum, steel, duralumin, and Inconel. The target workpieces range from small structures with complex shapes, such as springs and gears, to large structures, such as aircraft wings and jet turbines. Because the optimum machining parameters such as shot diameter and velocity differ depending on the target workpiece, it is necessary to clarify the physical phenomena and develop a technology that enables the search for the optimum machining conditions.

Previous studies on shot peening have reported the effects of shot velocity, diameter, and angle of attack on the residual stress, surface roughness, and shot peening coverage distribution in the workpiece. Although there have been theoretical^1^ and experimental studies^2–5^, many studies using numerical analysis have been reported owing to the complexity of the phenomena and the large number of parameters.

Dynamic elastoplastic structural analysis was performed using commercial solvers based on the finite element method (FEM) to investigate the relationship between the shot parameters and physical phenomena occurring in the workpiece. Shivpuri et al.^6^ studied the relationship between the shot velocity and shot diameter of an S230 shot and residual stress profiles of ISI4340 workpieces in a single-shot condition. They also suggested that the optimum number of repeated shot impacts. Mann et al.^7^ investigated the impingement diameter and residual stress profile of AA2024-T351 workpieces using a coupled numerical nanoindentation technique. Asgari et al.^8^ used numerical analysis results and a response surface method to clarify the effects of shot diameter, shot velocity, number of impacts, and friction coefficient on the residual stress of AISI4340 workpieces. Xiao et al.^9^ investigated the effect of strain rate when a single shot impacts with a mild steel plate. They clarified that the craters and lips with the strain rate considered were smaller and shorter, respectively, than those without the strain rate considered. Meguid et al.^10,11^ studied the characteristics of residual stress and equivalent plastic strain under the conditions of multiple shots using symmetry boundary models. They suggested that the influences of shot velocity and separation distance between shots for those characteristics were very significant. Wang et al.^12^ studied the surface roughness at an impact location under multiple-shot conditions, where the impact location of the shot was random. Xiao et al.^13^ evaluated the influence of the number of collisions, the distance between collision points, and the density of collisions under the conditions of a single repeated shot colliding at the same location, double shots, and multiple shots, respectively. Ren et al.^14^ examined the residual stress profiles of TC4 Titanium workpieces under double-shot conditions by changing the shot velocity and the distance between collision points. He et al.^15^ investigated the relationship between surface roughness and shot velocity, shot diameter, shot angle of attack, and number of shots in multiple-shot conditions. Li et al.^16^ investigated the fatigue life of 2024-T351 aluminum alloy workpieces to determine the effect of crack growth rate and mechanism of crack growth using experiments and numerical analysis. Wang et al.^17^ studied the energy conversion when a single shot impacts. They clarified that the primary energy component input into the material is plastic deformation energy, and the rest is elastic deformation energy.

Furthermore, with improvements in computer performance and the development of the analysis model, the FEM solver is coupled with the discrete element method (DEM), where the shot is treated as a single discrete element, to dynamically analyze the motion of the shot. Murugaratnam et al.^18^ developed a DEM-FEM solver and studied the effects of shot velocity, shot diameter, and shot angle of attack on the residual stress profile. Tu et al.^19^ and Edward et al.^20^ studied the relationship between the residual stress and the restitution coefficient based on the impact and rebound velocity ratios. Bhuvaraghan et al.^21,22^ efficiently predicted the residual stress of Inco718 workpieces in the conditions of single and multiple shots. Li et al.^23^ studied the relationship between the shot angle of attack and the initial surface roughness of TC4 Titanium workpieces.

The effects of the jet stream from the nozzle were analyzed using computational fluid dynamics (CFD) by treating the shot as a two-phase flow model. Nguyen et al.^24^ clarified the relationship between the shot velocity and shot peening coverage. However, the residual stress and surface roughness were unclear because the structural analysis was ignored. Lin et al.^25^ investigated the residual stress of 18CrNiMo7-6 gear steel workpieces by varying the shot velocity, shot diameter, and machining process conditions. Although their analyses were performed in three dimensions, the effects of the jet stream were treated as a two-dimensional model. Therefore, three-dimensional effects, such as three-dimensional vortices and unsteady disturbances, are not sufficiently clear. When a particle collides and rebounds with the structural surface vortices are generated behind the particle and around the impact location depending on the fluid and particle conditions^26^. In the case of multiple particles, such as in shot peening, the flow field is more complex owing to these vortices and random motions of the particles^27^.

To simultaneously evaluate the random motions of particles and unsteady characteristics of the fluid, instead of treating the particles as discrete elements and using a Lagrangian analysis method such as DEM, it is necessary to treat the fluid and particles in the same domain as in the Eulerian analysis method and directly calculate the interaction between the object and fluid. Mittal et al.^28^ developed the immersed boundary method (IBM), which represents an object in the Eulerian fluid domain. Mizuno et al.^27^ developed a fluid-particle coupling solver based on the IBM and investigated the number of impacts, location of impacts, number of rebounding shots, and kinetic energy of the rebounding particles for the shot peening. The results of the two-way coupled analysis differ from those of one-way coupled analysis, suggesting that it was necessary to consider the effects of interaction between fluid and particle. Isoz et al.^29^ proposed a hybrid method of IBM and DEM to analyze flow-induced movements of interacting irregular particles. Chéron et al.^30^ analyzed a dense particle flow using IBM, which processes object boundary conditions according to the distance between particles. Studies using coupled IBM and FEM methods suggested that IBM is highly adaptive and robust to analyze fluid–structure interactions^31–33^.

In this study, multi-physics coupled simulations, in which a single shot collides with a workpiece, were performed using a developed fluid-particle-structure coupled solver to investigate the effects of shot impact velocity and shot diameter on the fluid, motion of the shot, and structure. This coupled analysis allows for the examination of fluid-particle-structure interactions, especially the relationships between the unsteady flow field, random motion of shot, and dynamic deforming structure. It would help to construct macroscopic models such as drag, coefficient of restitution, and contact models required for conducting large-scale shot peening simulations. The remainder of this study is organized as follows. The computational methods for the developed coupled solver are presented in Sect. “Computational method”. In Sect. “Validation of FEM solver”, to validate the accuracy of the developed structure solver, the residual stress and deformation of the structure when single-shot impacts were compared with previous experimental and numerical analysis results. In Sect. “Fluid-particle-structure coupling simulation”, during single shot falling, impacting, and rebounding, the characteristics of fluid, shot motion, and structure were investigated using the developed fluid-particle-structure coupled solver. The conclusions drawn from this study are presented in Sect. “Conclusion”.

## Computational method

### Fluid-particle-structure coupling

In the proposed coupled solver, the fluid-particle-structure coupling was handled using a loosely coupled method. This coupled solver was developed in-house in Fortran, and the parallel computing was performed using the MPI and OpenMP. The finite difference method was used for the fluid analysis to calculate an unsteady flow around a rigid particle. The particle and elastoplastic structure were represented by the immersed boundary method on the Cartesian grid. The finite element method was used for the structural analysis to calculate the elastoplastic material.

**Figure 1:**
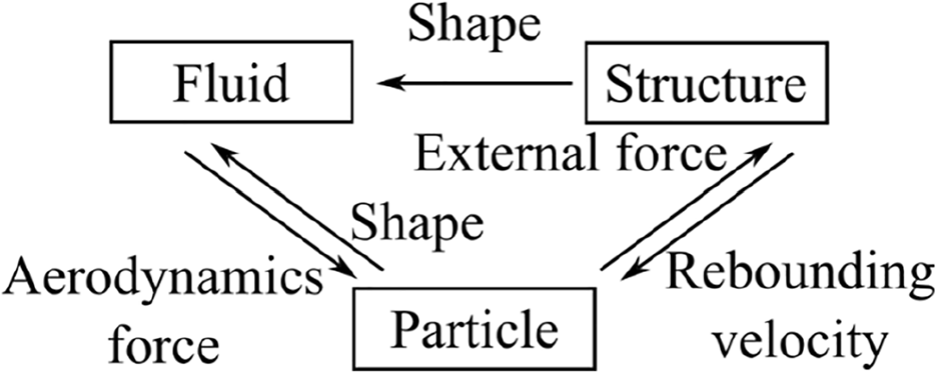
Coupling interactions of fluid, particle, and structure.

The proposed coupled solver directly evaluates the coupling interactions between; the fluid-particle, particle-structure, and structure-fluid, as shown in Fig. 1. In the fluid-particle interactions, aerodynamic forces calculated directly for the surface of the particle affect the particle velocity, and the boundary conditions of particle affect the surrounding pressure and flow velocity. This exchange of information is performed at each time step in fluid-particle analysis. The details of each computational method are described in Sects. “Fluid analysis” and “Particle analysis”. In the particle-structure interactions, the impact velocity and position of the particle pass on to the structure as external forces and contact surface position. Conversely, the structure conveys the rebound velocity to the particle based on the calculated reaction force. The details of each computational method are described in Sect. “Particle-structure contact model”. In structure-fluid interactions, the deformed structure surface affects the surrounding pressure and flow velocity by altering the boundary between flow fields and structure.

**Figure 2:**
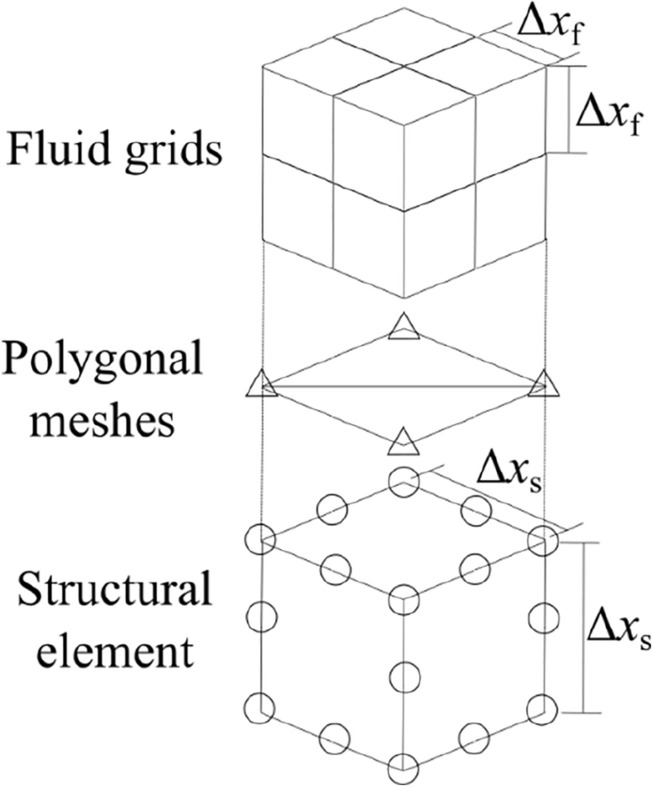
Fluid grids, polygonal meshes, and a structural element.

The computational domains were formed by the Cartesian grid and structural elements in the fluid and structural analyses, respectively. Polygonal meshes were used to exchange structural geometry information for each analysis, as shown in Fig. 2. The details of the object definition method are described in Sect “Fluid analysis”. The initial element size was set to *∆x*_s_ = 2*∆x*_f_, where *∆x*_f_ is the grid size, to arrange the node and grid point in the same location until the structure deformed. The information in the fluid grid is exchanged directly with the structural node at the same location.

**Figure 3:**
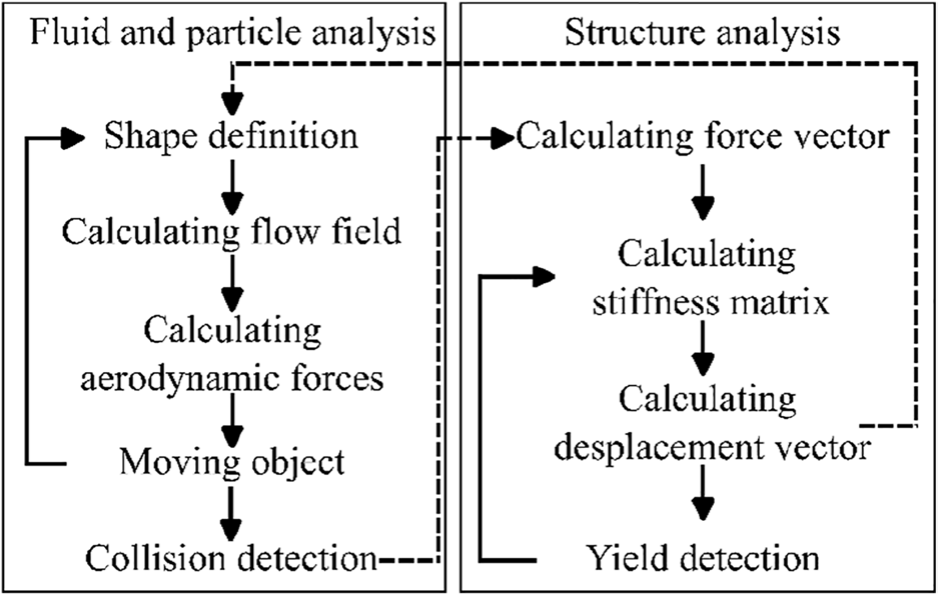
Flowchart of coupled simulation.

The time increment in the flow simulation was calculated based on the maximum velocity of the fluid and particle at each step. The structural simulation was performed when the particle collided with the structure and was repeated several times during the one step of the flow simulation. The number of time steps and time increments of the structural simulation were defined based on the particle-structure contact time and the Courant number of the structural analysis. Finally, Fig. 3 shows a flowchart of the coupled simulation.

### Fluid analysis

The governing equations are the three-dimensional incompressible Navier–Stokes equations and the equations of continuity.

1 $$\nabla \cdot \mathbf{V}=0$$

2 $$\frac{\partial \mathbf{V}}{\partial t}+\mathbf{V}\left(\nabla \cdot \mathbf{V}\right)=-\frac{1}{\rho }\nabla p+\nu {\nabla }^{2}\mathbf{V}$$

where **V** = (*u*_f_, *v*_f_, *w*_f_) is the vector components of fluid velocity, and *p*, *ρ*, and *ν* are the pressure, density of the fluid, and kinematic viscosity, respectively. The mesh employed was an equally spaced three-dimensional Cartesian grid. The fractional step method was applied to the time marching. The convection term was evaluated using a second-order skew-symmetric scheme^34^ and the diffusion term was discretized using a second-order central-difference scheme. The Poisson equation of pressure was calculated using the successive over-relaxation (SOR) method. Validation of the analysis using these methods has been conducted in previous studies^27^.

In fluid analysis, objects are represented by level set and ghost-cell methods^27,28,35^. The level set function *φ* is defined as the signed minimum distance between whole cells and the object boundary. The movement or deformation of multiple objects is solved using multiple-level set functions based on a simple minimum-distance approach^36^. The level set function of the deforming object is calculated by the inside/outside determination of the object using the Möller-Trumbore intersection algorithm based on the information of the polygon mesh. The surface topography of the structure can be evaluated smoothly because the object is represented by a level set function. Each cell is classified as a fluid cell (FC), ghost cell (GC), or object cell (OC) using the level set function:

3 $$0<{\phi }_{\text{FC }}-2.25\Delta x\le {\phi }_{\text{GC}}\le 0 {\phi }_{\text{OC}}<-2.25\Delta x$$

This ghost cell is used to calculate the boundary conditions on the object surface and maintain robustness for calculating the motion of the object and pre/post-impact. The aerodynamic pressure and friction forces acting on the object surface were calculated for the cell face between the fluid and ghost cells. In the proposed method, the forces were estimated using a simple algorithm without surface polygons^37^.

### Structural analysis

Structural elements were evaluated using the 20-nodes iso-parametric rectangular elements. The elastic–plastic stress–strain relations were defined by the Prandtl–Reuss equations. The failure criterion was based on the von-Mises criterion. The governing equations using matrices are stiffness equations including the inertial force as follows:

4 $$\left[M\right]\frac{{d}^{2}}{d{t}^{2}}{\mathbf{X}}_{\text{s}}+\left[K\right]{\mathbf{X}}_{\text{s}}=\mathbf{F}$$

where [*M*], [*K*], **X**_s_ = (*x*_s_, *y*_s_, *z*_s_), and **F** = (*f*_x_, *f*_y_, *f*_z_) are the lamped mass, global stiffness matrices, global displacement vector, and external force vector respectively. Equation (4) is calculated using the second-order accurate central-difference scheme.

The Johnson–Cook equation^38^ was employed to evaluate the stress-plastic strain relationship as follows:

5 $$\sigma =\left(A+B{\upvarepsilon }^{n}\right)\left(1+C\mathit{ln}\frac{\dot{{\upvarepsilon }^{*}}}{{\upvarepsilon }_{0}}\right)\left(1-{\left(\frac{T-{T}_{0}}{{T}_{m}-{T}_{0}}\right)}^{m}\right)$$

where *σ* is the equivalent stress. *ε*, ε^*^and *ε*_0_ are equivalent plastic strain, applied strain, and reference strain. *T*, *T*_0_, and *T*_m_ denote the applied, reference, and melting temperatures, respectively. *A*, *B,* and *C* are the initial yield strength, strain-hardening constant, and strengthening coefficient of the strain rate, respectively. *n* and *m* denote the coefficients of strain hardening and thermal softening, respectively. In this study, the value of *T* was the same as *T*_0_^15^.

### Particle analysis

Particle movement is described by the equations of motion for *xyz*-transportations of a rigid body as follows:

6 $$\frac{d\left({m}_{\text{p}}{\mathbf{u}}_{\text{p}}\right)}{dt}={\int }_{S}fdS+{m}_{\text{p}}\mathbf{g}$$

7 $$\frac{d{\mathbf{X}}_{\text{p}}}{dt}={\mathbf{u}}_{\text{p}}$$

where *m*_p_ denotes the particle mass. **u**_p_ = (*u*_p_, *v*_p_, *w*_p_) and **X**_p_ = (*x*_p_, *y*_p_, *z*_p_) represent the velocity and position of the center of mass of the particle, respectively. *f* expresses the aerodynamic forces extended on the particle by the fluid. *S* is the cell face between the fluid and ghost cells, and **g** denotes the acceleration owing to gravity. Equations (6) and (7) are calculated using Runge–Kutta methods. The distribution of the level set function changes with the particle movement. The velocity and pressure components of the ghost cell were not updated when an image point appeared in the ghost cell of another object.

### Particle-structure contact model

The external force distribution and object collision time are generally defined using the Hertz theory model. However, depending on the element width, the stress distribution was not defined within each element. Therefore, the developed solver adopted a rigid-body contact model. The displacement of the structure was evaluated using forced displacement based on the shape of the particle. The distance between the node position of the surface structure and the center position of the particle *R*_p_ is given by

8 $${R}_{\text{p}}=\sqrt{{x}_{\text{p}}^{2}+{y}_{\text{p}}^{2}+{z}_{\text{p}}^{2}}$$

where *x*_p_, *y*_p,_ and *z*_p_ are the pre-modified surface positions. When the particle is assumed to be a rigid body, *R*_p_ becomes greater than the particle radius *R*. If the particle collides with the surface of the structure, *R* is given by

9 $$R=\sqrt{{x}^{2}+{y}^{2}+{z}^{2}}$$

where *x*, *y,* and *z* are the post-modified positions. When the movement of the particle becomes smaller than *R*, horizontal movement can be neglected. The displacement is modified only in the vertical direction.

10 $$R=\sqrt{{x}_{\text{p}}^{2}+{y}_{\text{p}}^{2}+{z}^{2}}$$

Finally, the post-modified position for the vertical direction is given by

11 $$z=\sqrt{{R}^{2}-{R}_{\text{p}}^{2}+{z}_{\text{p}}}$$

**Figure 4:**
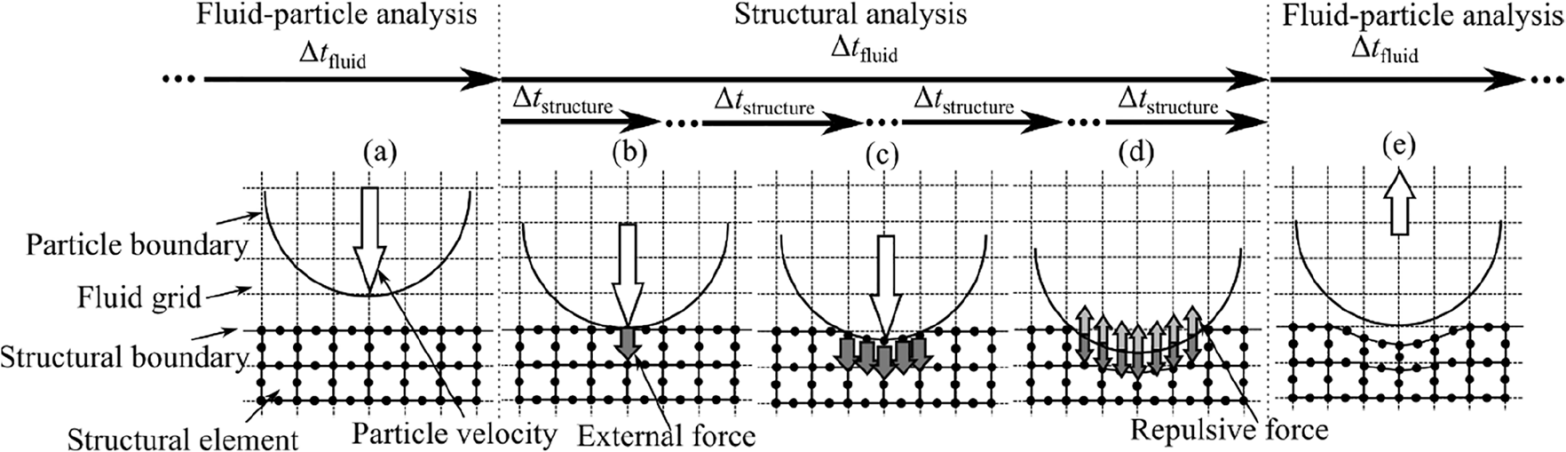
Schematic of the collision procedure.

Figure 4 shows the schematic of the collision procedure, where Δ*t*_fluid_ and Δ*t*_structure_ are the time increments of fluid and structural analyses, respectively. In Fig. 4a, the behavior of the particle is calculated by the fluid and particle motion coupled analysis. The collision detection of particles and structures is determined using an inequality involving Pythagoras' formula without the margin of gaps between objects. When the particle contacted with the surface of the structure, the structural analysis is initiated, as shown in Fig. 4b. In structural analysis, impact forces are not calculated directly. Once contact between the particle and the structure occurs, the impact force is calculated by performing a structural analysis based on the displacement of the particle shape, which is considered to be transferred to the structural surface when the particle penetrates the surface. The impact force is used to modify the particle velocity, and the displacement of the particle when it advances one structural time step can be used to determine the displacement of the structural surface again. By repeating these procedures, the deformation of the structural surface and the change in particle velocity can be obtained sequentially, as shown in Fig. 4c. Impact forces are applied to the nodes with which the surfaces are in contact. The distribution of the impact force on the nodes is determined spherically by the positional relationship before and after displacement obtained from Eq. (11). In Fig. 4d, the state in which the structure satisfies the yield condition and impact forces are balanced is calculated. The particle rebounds by the repulsive forces from the structure. The rebounding behavior of the particle is again calculated by the fluid and particle motion coupled analysis, as shown in Fig. 4e. It was confirmed that the sum of the kinetic energy of the particle and the internal energy of the structure before and after the collision is conserved. The frictional and thermal effects were excluded in this contact model.

## Validation of FEM solver

### Computational conditions

To verify the accuracy of the proposed FEM solver using the developed rigid body contact model, the residual stress and displacement were compared with the experimental and numerical analysis results obtained by Kobayashi et al.^2^ and Shivpuri et al.^6^. These models were compared using a dynamic impact test in which a single shot collided with the steel plate. The shot was a steel ball with the diameter *D*_p_ = 50 mm and was set to an impact velocity *w*_p,impact_ = 6.3 m/s. The initial shot location was set so that the shot and the structure surface were nearly in contact. In addition, the use of experimental values for the initial shot velocity allows the effects of the flow field and gravity to be ignored in the structural validation since the experimental shot velocity near the structure surface already includes the effects of the fluid flow and gravity. The chemical composition of the steel plate was treated as JIS S48C in the experiment by Kobayashi et al., and AISI4340 in the numerical analysis by the proposed model and Shivpuri et al. The chemical compositions of JIS S48C and AISI4340 were similar, as summarized in Table 1.

**Table 1:** Chemical compositions of JIS S48C and AISI 4340.

Chemical composition wt%	C	Si	Mn	P	S
JIS S48C	0.45–0.51	0.15–0.35	0.6–0.9	< 0.030	< 0.035
AISI 4340	0.38–0.48	0.15–0.30	0.6–0.8	< 0.035	< 0.040

The computational domain measured 100 × 100 × 50 mm. The three different initial elemental sizes were fixed at 1.25, 2.00, and 2.50 mm, and the number of elements is located at 80 × 80 × 40, 50 × 50 × 25, and 40 × 40 × 20, respectively. In structure analysis, boundary conditions were applied such that the normal displacement was zero for the *x* and *y* directions, and all displacements were zero at the bottom of the structural domain. In Table 2, the mechanical properties and the parameters of the Johnson–Cook model are listed.

**Table 2:** Mechanical properties and parameters of the Johnson–Cook model.

Density kg/m^3^	Elasticity modulus GPa	Poisson’s ratio	*A* MPa	*B* MPa	*n*	*C*	*ε*_0_
7800	205	0.29	1498	943.8	0.26	0.014	1

### Computational results

**Figure 5:**
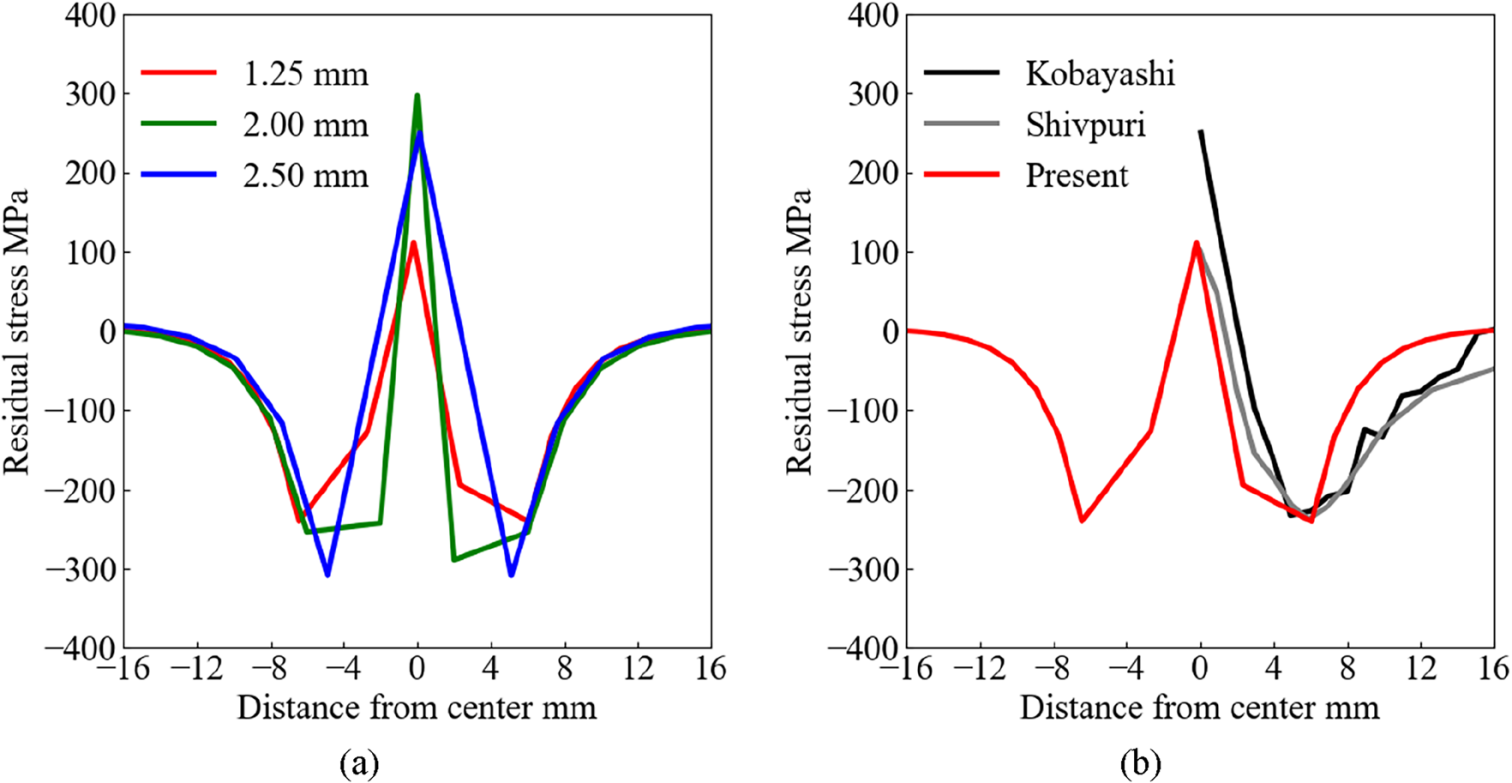
Residual stress at the surface of structure: (**a**) comparison by element size, and (**b**) comparison with previous studies.

Figure 5a and b show the residual stress at the contact surface of the structure. The horizontal and vertical axes indicate the distance from the impact point and residual stress obtained from the surface elements, respectively. Figure 5a shows the results obtained from three different element sizes with red (1.25 mm), green (2.00 mm), and red (2.50 mm) lines. The results obtained from the experiment by Kobayashi et al. and from the numerical analysis using FEM by the proposed model and Shivpuri et al. are shown in Fig. 5b. The previous experimental and numerical results are presented for the horizontal axis range of *x* = 0–16 mm. Each result captures characteristics and trends. Tensile residual stress occurs at the center of the impact, compressible residual stress is generated away from the center point, and residual stress gradually approaches zero Pa above *x* = 5 mm. The different element sizes qualitatively captured the trends, as shown in Fig. 5a. The results of this study are in good agreement with previous experimental and numerical results, as shown in Fig. 5b. The difference in residual stress could be attributed to the different chemical compositions and Johnson–Cook model.

**Figure 6:**
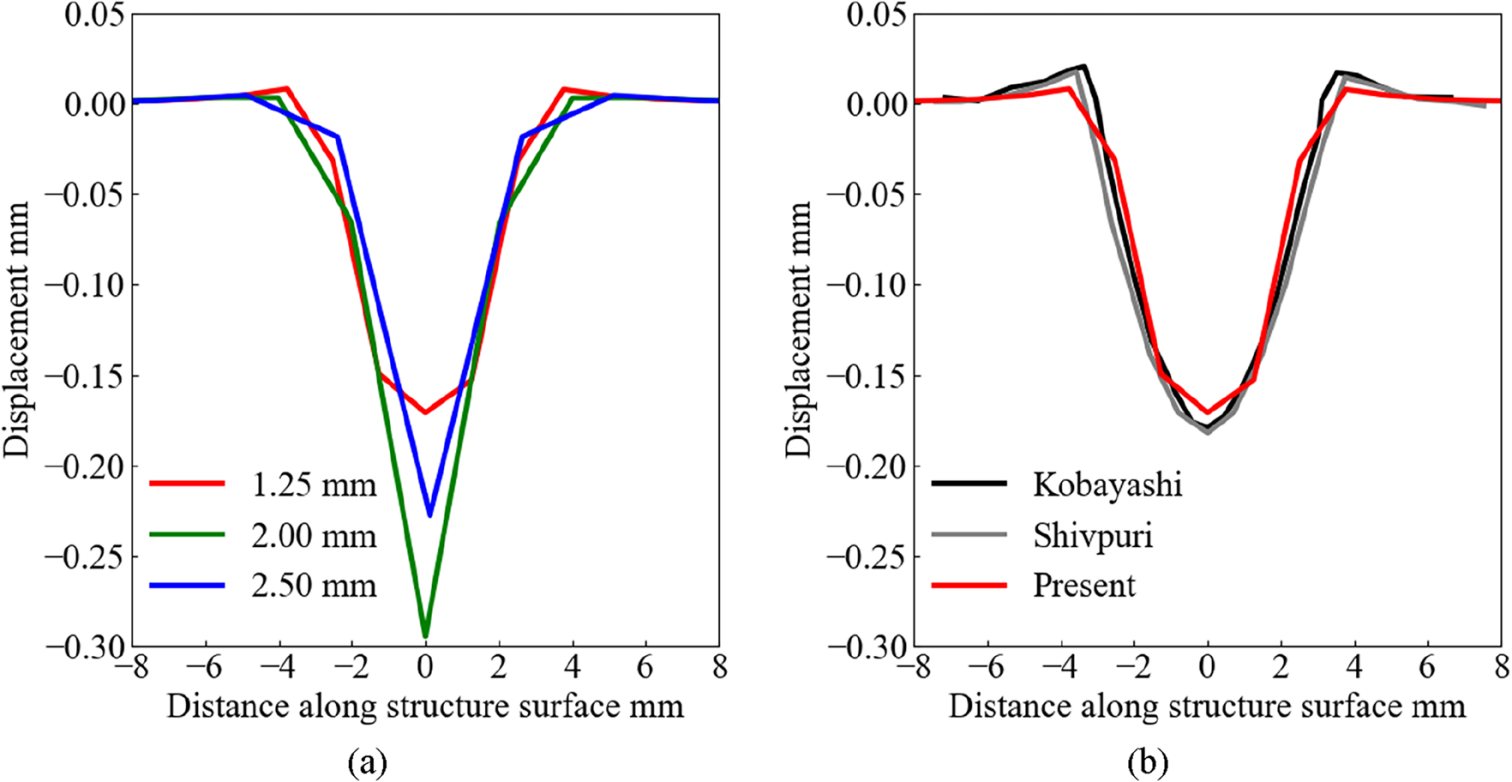
Indentation profile at the surface of structure: (**a**) comparison by element size, and (**b**) comparison with previous studies.

Figure 6a and b show the indentation profiles of the contact surface of the structure. The horizontal and vertical axes indicate the distance from the impact point and displacement from the surface, respectively. The previous experimental and numerical results are presented in the horizontal axis range of *x* = 0–8 mm. The displacement was largest at the center point, and the impact crater edge was created at *x* = 4 mm. The different displacement results were caused by the different element size conditions, as shown in Fig. 6a. The present result is in good agreement with previous experimental and numerical results, as shown in Fig. 6b.

## Fluid-particle-structure coupling simulation

### Computational conditions

A coupling analysis considering the flow field around a single shot impacting an elastoplastic structure was performed using the proposed fluid-particle-structure coupling solver. The effects of uniform flow velocity and shot diameter on the pre- and post-collision phenomena were examined.

The coupled analysis was performed in a series of computational processes as follows. First, the steady flow was calculated as the initial flow field condition, excluding shot. When the flow field is formed, shot with the same velocity as the uniform flow intruded into the computational domain from the outside. Second, the unsteady flow field around the falling shot was analyzed. Subsequently, the shot moved under aerodynamic forces and collided with the structure. When the shot contacts the structure, dynamic structural analysis is conducted until it rebounds. Finally, the unsteady flow fields around the rebounding shot and deformed structure are computed.

**Figure 7:**
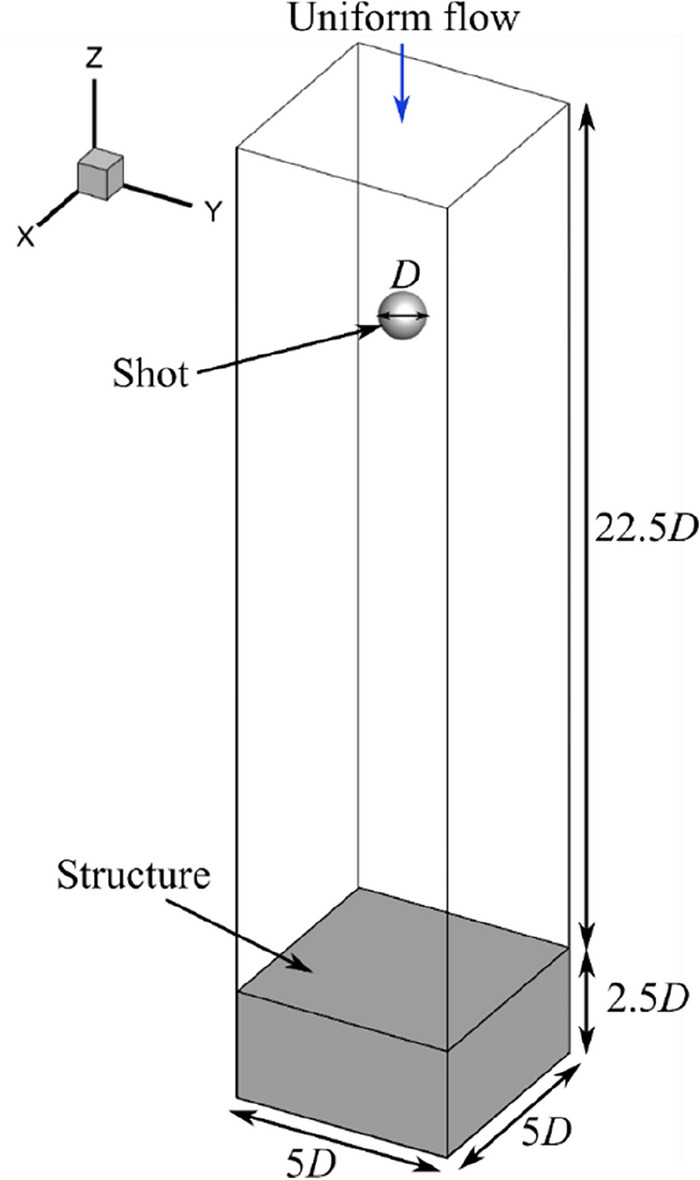
Computational model of coupling simulation.

The computational conditions were defined based on the shot diameter *D* and uniform flow velocity *v*_*f*_. The shot diameter and initial shot velocity (= uniform flow velocity) were set to *D* = 0.2, 0.4, 0.5, 0.8, and 1.0 mm with three velocities for each diameter condition, as listed in Table 3. The relative Reynolds number Re^*^ =|*v*_*f*_—*v*_*p*, after_|*D* / *ν* should be defined from the relative velocity obtained from the uniform flow velocity and the rebounding shot velocity *v*_*p*, after_, the kinematic viscosity coefficient *ν*. However, the rebounding shot velocity *v*_*p*, after_ is unknown, then the Reynolds number *Re* =|*v*_*f*_ |*D* / *ν* is determined from the uniform flow velocity and computational conditions are set at five Reynolds numbers. The density and viscosity of the flow field were set to 1.205 kg/m^3^ and 1.822 × 10^−5^ Pa∙s, respectively. The shot was a rigid steel sphere with a density of 7,800 kg/m^3^. The height of the flow field was the same as that used in a previous study^27^. In the structural analysis, the chemical composition of the steel plate was treated as AISI4340 and applied the parameters listed in Table 2. The computational domain of fluid analysis was 5*D* × 5*D* × 22.5*D*, which consisted of the region of structure 5*D* × 5*D* × 2.5*D* and flow field 5*D* × 5*D* × 20*D*, as shown in Fig. 7. The initial position of the shot was fixed at (*x*, *y*, *z*) = (2.5*D*, 2.5*D*, 23.5*D*). The number of grids for the fluid analysis and elements of the structural analysis are arranged as 200 × 200 × 900 and 100 × 100 × 50, respectively. In fluid analysis, the inlet boundary values of velocity and pressure were defined by Dirichlet and Neumann boundary conditions, respectively, and all variables are subject to Neumann conditions at other boundaries. In structure analysis, boundary conditions were applied such that the normal displacement was zero for the *x* and *y* directions, and all displacements were zero at the bottom of the structural domain.

**Table 3:** Computational cases of coupling simulations.

Case	Diameter mm	Velocity m/s	Reynolds number
D02V20	0.2	20	235
D02V40	0.2	40	532
D02V80	0.2	80	1063
D04V20	0.4	20	532
D04V40	0.4	40	1063
D04V80	0.4	80	2126
D05V16	0.5	16	532
D05V32	0.5	32	1063
D05V64	0.5	64	2126
D08V10	0.8	10	532
D08V20	0.8	20	1063
D08V40	0.8	40	2126
D10V16	1.0	16	1063
D10V32	1.0	32	2126
D10V64	1.0	64	4252

The validation of the influence of computational grid size on the random motion of the shot after the impact is summarized in Appendix 1.

### Flow field and relative reynolds number

**Figure 8:**
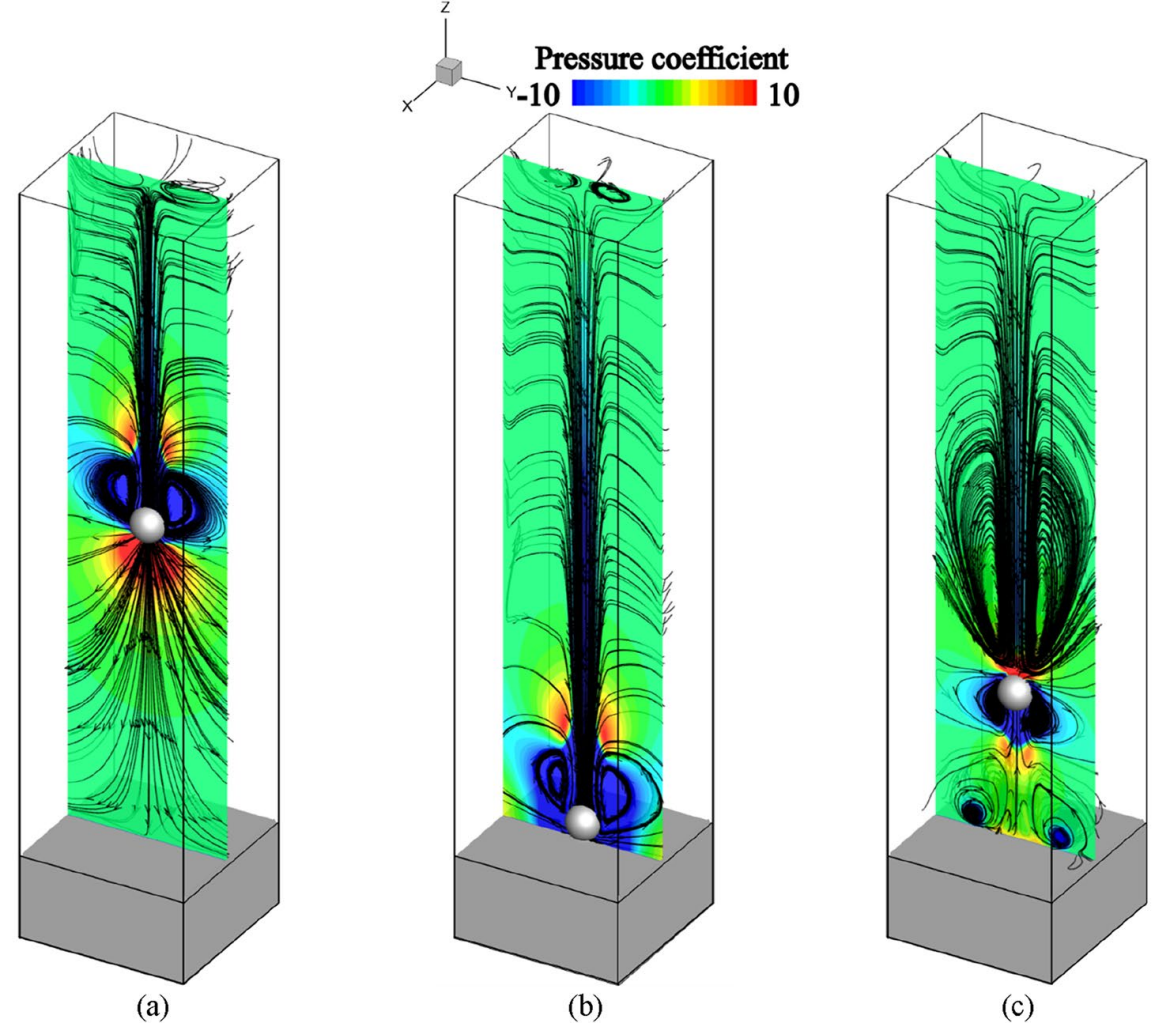
Instantaneous distributions of pressure coefficient with streamlines in case of D10V16: (**a**) with falling shot at t^*^ = 11.5 (**b**) with impacting shot at t^*^ = 20.5, and (**c**) with rebounding shot at t^*^ = 27.5.

Figure 8 shows the instantaneous distributions of the pressure coefficient with streamlines at the center of the computational domain for D10V16 at non-dimensional times t^*^ = 11.5, 20.5, and 27.5, based on the uniform flow velocity and shot diameter. By this normalization, the shots with different conditions can reach the surface at t^*^ = 20. When the shot falls with the freestream at t^*^ = 11.5 as shown in Fig. 8a, wake vortices are generated in the shot wake. When the shot collides with the surface of the structure at t^*^ = 20.5, as shown in Fig. 8b, the flow field develops near the surface of the structure. The rebounding shot thrusts forward through the freestream and the vortices formed as the shot fell at t^*^ = 27.5, as shown in Fig. 8c. The flow field further developed because a vortex ring was generated near the surface of the structure.

**Figure 9:**
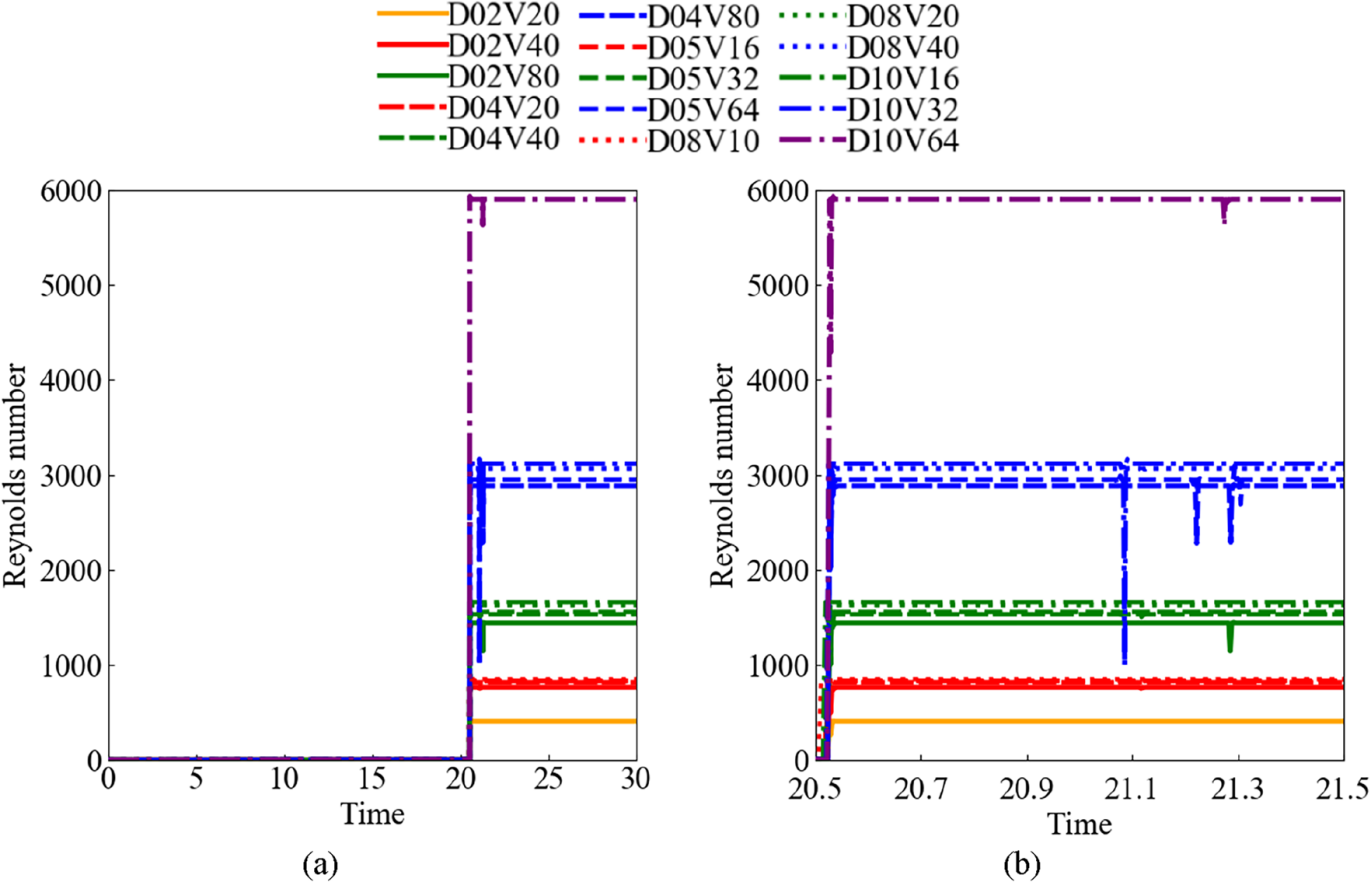
Non-dimensional time histories of relative Reynolds number: (**a**) at the time t^*^ = 0–30 of the pre-/ post-collision, and (**b**) at the time t^*^ = 20.5–21.5 of the post-collision.

Figure 9 shows a time history of the relative Reynolds number. The horizontal axis represents the non-dimensional time based on the uniform flow velocity and shot diameter. The vertical axis represents the relative Reynolds number Re^*^ based on the relative velocity obtained from the uniform and shot velocities along the *z*-axis. The line color and line are distinguished by the Reynolds number *Re* and shot diameter *D*. At t^*^ = 0–20, the relative Reynolds number is zero because the shot velocity was the same as the uniform flow velocity as shown in Fig. 9a. At t^*^ = 20.5, the shot collided with the structural surface. After t^*^ = 20.5, the relative Reynolds number was almost constant; however, it increased and then decreased rapidly from t^*^ = 21.09–21.31 in high-velocity cases of the same relative Reynolds number conditions, respectively. The relative Reynolds number was mostly the same under the same Reynolds number conditions. Therefore, when the flow field and shot conditions are defined by the Reynolds number, the flow field after the collision can be organized using the relative Reynolds number. In high-relative Reynolds number cases, the sharp fluctuation in the relative Reynolds number was caused by changes in the shot motion behavior. As discussed below, this shot motion behavior is affected by the unsteady flow field and large deformations of the structure.

### Shot velocity and coefficient of restitution

**Figure 10:**
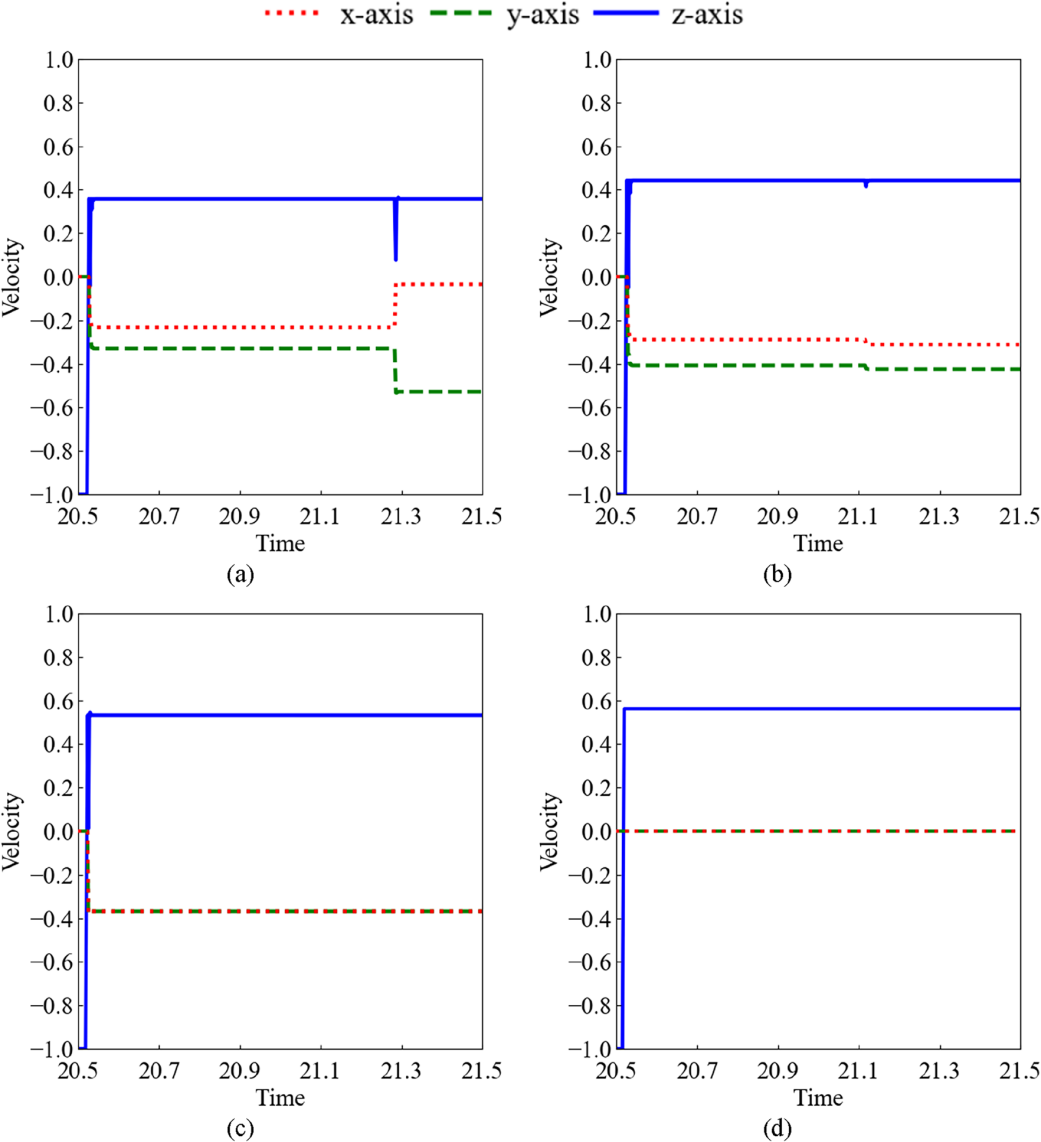
Non-dimensional time history of normalized shot velocity: (**a**) D02V80 (b) D04V40, (**c**) D08V20, and (**d**) D10V16.

Figure 10 shows a time history of the shot velocity after the shot impacts. In Fig. 10a–d, the results for (a) D02V80, (b) D04V40, (c) D08V20, and (d) D10V16 are shown, and the relative Reynolds number is almost Re^*^ = 1600. The horizontal and vertical axes represent the non-dimensional time and non-dimensional velocity normalized by the uniform flow velocity. The lines represent the velocity components along each axis. Before the shot collided with the structure surface, the velocity components of the *x*-axis and *y*-axis directions (red dots and green dash lines) are zero and the *z*-axis (blue line) velocity component is -1.0. The results of the following four characteristics are shown for the velocity components of the *x* and *y* directions. As shown in Fig. 10 for (a) D02V80, the velocity components of the *x*-axis and *y*-axis directions occur after the collision. Thereafter, each component remained constant and fluctuated significantly simultaneously with the *z*-axis velocity. Figure 10 for (b) D04V40 shows a case in which the fluctuations are small. The case without fluctuations is shown in Fig. 10 for (c) D08V20. The velocity components of the *x* and *y* directions were also generated after the collision in the cases of D04V40 and D08V20, respectively. As shown in Fig. 10 for (d) D10V16, after collision, the velocity components of the *x*- and *y*-axes did not fluctuate. These fluctuations also affected the variability of the relative Reynolds number as shown in Fig. 9b. In other relative Reynolds number conditions, the characteristics such as shown in Fig. 10a were obtained under the high-velocity impact conditions. As the impact velocity decreases, the characteristics change from Fig. 10b–d.

**Figure 11:**
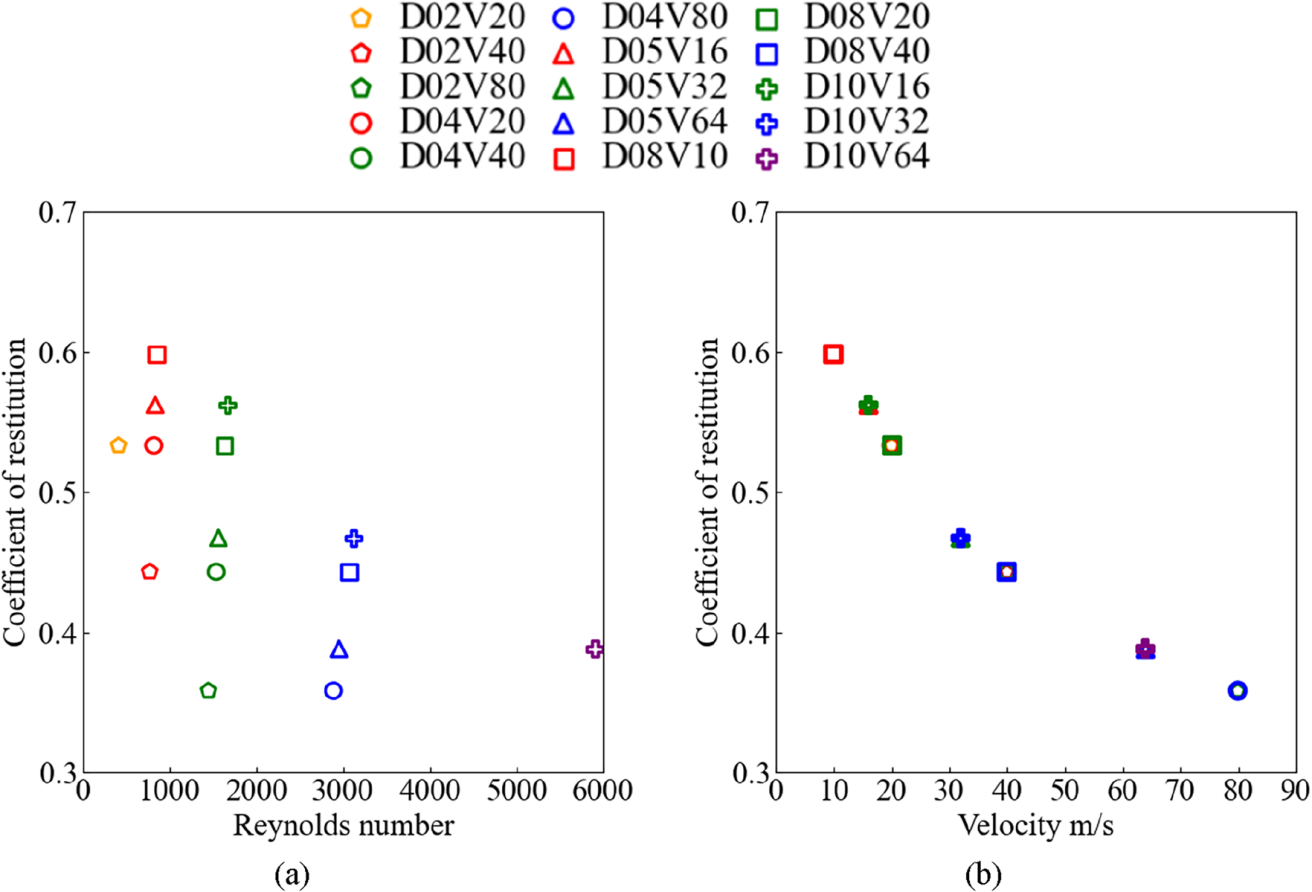
Coefficient of restitution: (**a**) relative Reynolds number, and (**b**) uniform flow velocity.

The change in the kinetic energy of the shot before and after the collision is directly captured by coupled particle-structure analysis. The shot rebounds with the *z*-axis velocity component decelerated after the collision, as shown in Fig. 10. Figure 11 shows the relationship between the coefficient of restitution for (a) the relative Reynolds number and (b) uniform flow velocity. The coefficient of restitution was the same for varying shot diameters at the same velocity, as shown in Fig. 11a. However, for the same shot diameter, the coefficient of restitution decreased as the uniform flow velocity increased. Under the conditions of the present analysis, the uniform flow velocity had a stronger effect on the restitution coefficient than the shot diameter, as shown in Fig. 10b. As the coefficient of restitution was different in each case, the *z*-axis velocity components after the collision yielded different results for each case, as shown in Fig. 10.

### Residual stress and deformation of the surface of the structure

**Figure 12:**
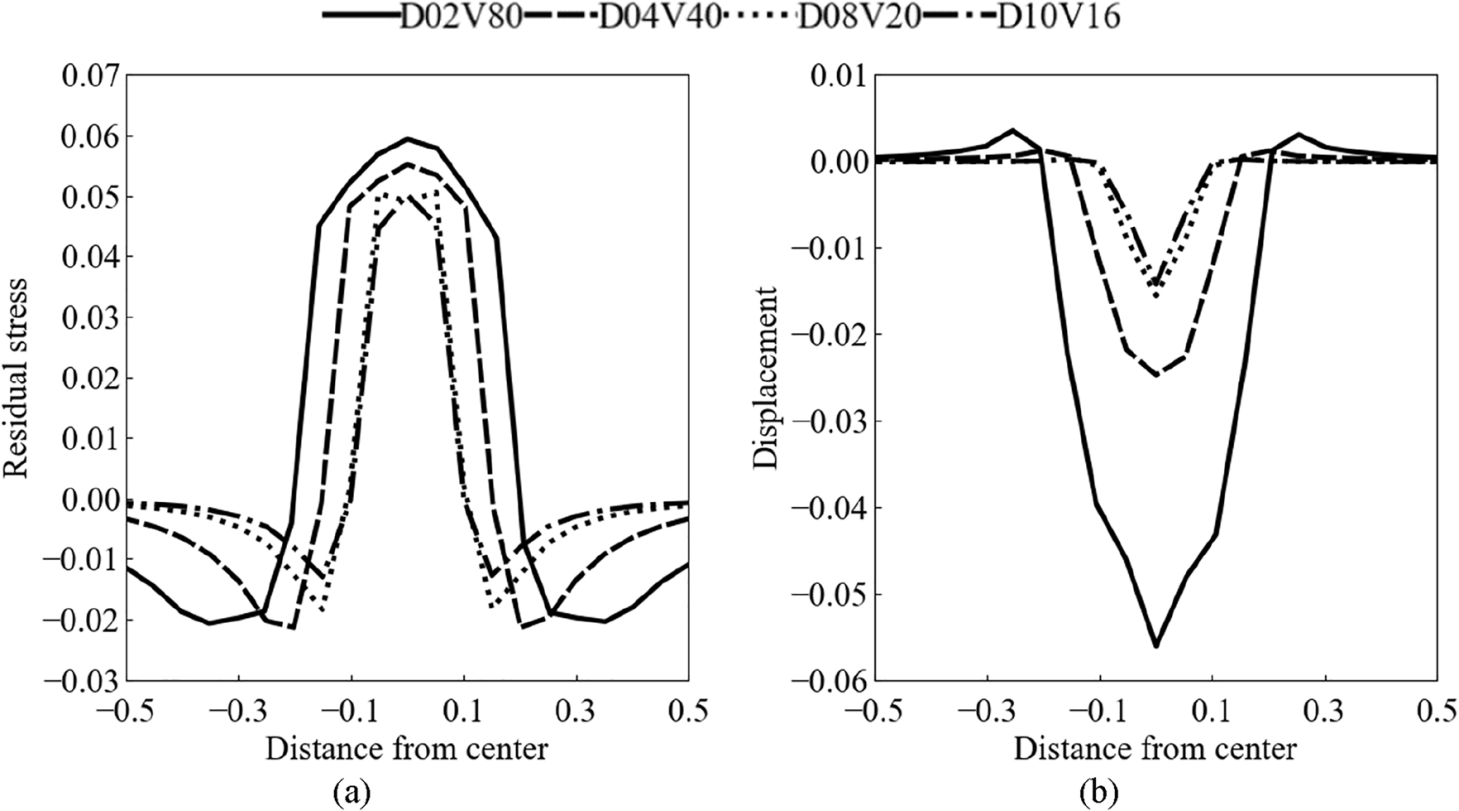
Instantaneous distributions at the surface of structure in case of Re^*^ = 1600: (**a**) residual stress, and (**b**) indentation profile.

The variation in the kinetic energy of the shot before and after the collision is related to the residual stress generated in the structure owing to the collision. Figure 12 shows the instantaneous distribution of the residual stress and indentation profile at the surface of the structure after the collision in the case of Re^*^ = 1600. Figure 12a shows the distribution of the non-dimensional residual stress normalized by the elasticity modulus at the surface of the structure. The horizontal axis indicates the distance from the impact point normalized by the shot diameter. Under the same Reynolds number conditions, the residual stress shows different distributions. The maximum residual stress at the impact center location increased in case of high shot velocity and small shot size. The relationship between shot velocity and residual stress is consistent with the trend in the coefficient of restitution shown in Fig. 11. Figure 12b shows the indentation profile normalized by the shot diameter at the surface of the structure. The horizontal axis indicates the distance from the impact point normalized by the shot diameter. The sizes of the depression and impact crater edges increased as the shot velocity increased and shot size decreased. The residual stress and normalized indentation profiles were asymmetrically distributed for the impact center location. In other conditions for the relative Reynolds number, the same trend was qualitatively observed, and the magnitude of residual stress and deformation changed as the relative Reynolds number increased or decreased. The tendency to form for the asymmetric indentation increased under conditions with the relative Reynolds numbers and the impact velocities exceeded 1600 and 40 m/s, respectively. These residual stress distributions and indentation profiles are similar to the results shown in Figs. 5 and 6, indicating that the proposed coupled solver accurately captures structural features.

### Shot behavior due to asymmetrically fluid and structure distributions

**Figure 13:**
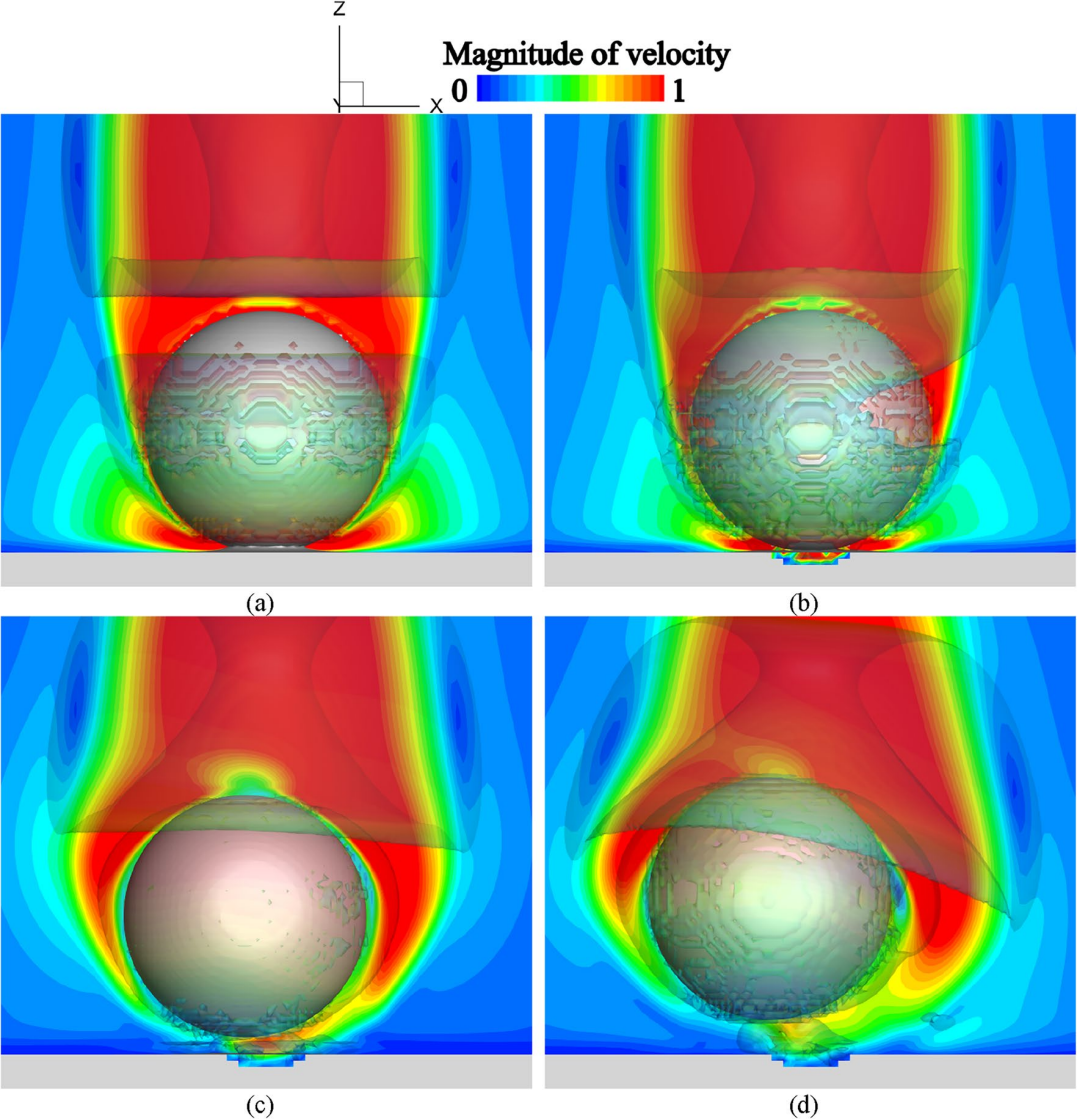
Instantaneous distributions of the magnitude of velocity and second invariant of velocity tensor in case of D02V80: (**a**) at t^*^ = 20.52, (**b**) at t^*^ = 20.53, (**c**) at t^*^ = 20.90, and (**d**) at t^*^ = 21.25.

In the case of D02V80, the shot collides with the surface of the structure and the shot rebounds in the *x*- and *y*-axes directions as shown in Fig. 10a. Moreover, the shot changes its traveling direction in the *x*- and *y*-axes directions at t^*^ = 21.25. Figure 13 shows the instantaneous cross-sectional distributions of the magnitude of the velocity normalized by the uniform flow velocity, and the second invariant of the velocity tensor in the case of D02V80. The isosurfaces of objects are visualized as staircases in the visualization but are represented smoothly in the computation by the level set functions. The flow field was symmetrically formed at the instant at which the shot impacted the surface of the structure, as shown in Fig. 13a. As shown in Fig. 13b, the flow field was asymmetrically formed around the shot and inside the cavity owing to the asymmetric concavity of the structure. Consequently, the shot is subjected to nonuniform aerodynamic forces, and the shot moves in the *x*- and *y*-axes directions. The proposed coupled solver can robustly analyze the collision between objects and moving/deforming objects. The flow distributions are visualized at t^*^ = 20.90 and t^*^ = 21.25 when the velocity components of the *x*- and *y*-axes directions change as shown in Fig. 13c and d. The shot moved to the front side of the cross-section. The tilted motion of the shot was caused by the asymmetric flow field and complex vortex structures around the shot as shown in Fig. 13c. The vortices were created by the flow between the depression created by the collision and the rebounding shot. When the shot reached the impact crater edge at t^*^ = 21.25 as shown in Fig. 13d, the influence of the asymmetric flow field increased because of the change in the distance between the shot and surface of the structure. Moreover, the vortices became even larger and more complex. Consequently, the shot changed direction again owing to non-uniform aerodynamic forces. These results suggest that the motion of the shot was affected by the unsteady flow field created by the collision. Furthermore, the direction of their motion depends on the asymmetry and size of the depression and the impact crater edge. Such asymmetric phenomena are difficult to reproduce without the coupled analysis because different combinations of impact velocity and shot diameter change the elastoplastic deformation process under the same relative Reynolds number conditions. Strong randomness appeared in the shot behavior under the relative Reynolds number above 1600 and the impact velocity above 40 m/s, where flow disturbance and crater edges became more significant.

## Conclusion

Using the developed fluid-particle-structure coupled solver, the characteristics of the fluid, motion of the shot, and elastoplastic structure were investigated in the case of a falling, impacting, and rebounding single shot. The residual stress and indentation profiles simulated by the structure solver using the developed rigid body contact model were compared with those of previous studies on the collision between a single steel shot and an AISI4340 plate. As a result, the residual stress and indentation profiles at the surface of the structure obtained from the present results are in good agreement with those obtained in the previous studies. The relationship between uniform flow velocity and shot diameter was examined under conditions based on the Reynolds number using the developed coupled solver. The main results obtained by the coupled analysis that directly evaluated the coupling interaction are summarized below.

1. The proposed coupled solver can accurately and robustly analyze the physical characteristics of the fluid, moving particle, and structure.
2. The post-collision flow field can be characterized by the relative Reynolds number based on the shot diameter and relative velocity between the uniform flow and rebounding shot velocities.
3. In the absence of the fluid analysis, the residual stress and indentation size are known to increase due to the significant impact energy and momentum as the impact velocity and shot size increase. In the coupled analysis, in contrast, the residual stress and indentation size were increased as the relative Reynolds number increased. On the other hand, the coefficient of restitution decreased due to the larger residual stress remaining in the structure. Moreover, the tendency to form the asymmetric indentation was enhanced under conditions with the relative Reynolds numbers above 1600 and the impact velocity above 40 m/s. This suggested that the impact dynamics due to the flow field and motion of the shot are more complex than without the coupled analysis, leading to the non-uniform residual stress and indentation.
4. Due to the asymmetric indentation of the structure, the flow field was asymmetrically formed, and the shot rebounded obliquely by non-uniform aerodynamic forces. Such asymmetric phenomena are difficult to reproduce without the coupled analysis because different combinations of impact velocity and shot diameter change the elastoplastic deformation process under the same relative Reynolds number conditions.
5. The random behavior of the shot after the collision was affected by the states of the flow field and the structure. This was particularly affected by the asymmetric vortices and surface topography of the structure. Strong randomness appeared under conditions with relative Reynolds numbers above 1600 and the impact velocity above 40 m/s, where flow disturbance and crater edges are more significant.

The obtained results from coupled simulations, when performing the analysis for actual shot peening conditions, suggested the need to evaluate not only the dynamic analysis of the structure but also the unsteadiness of the flow field and random motion of the shot simultaneously. Such knowledge, in conjunction with experimental results, might help to construct macroscopic models such as drag, coefficient of restitution, and contact models required for conducting large-scale shot peening simulations. Further studies on multi-shot conditions are required, in which a more complex flow field, random motion of the shot, and multi-indentation occur owing to the collision between the shots or shot and structure.

## Figures and Tables

**Figure 14:**
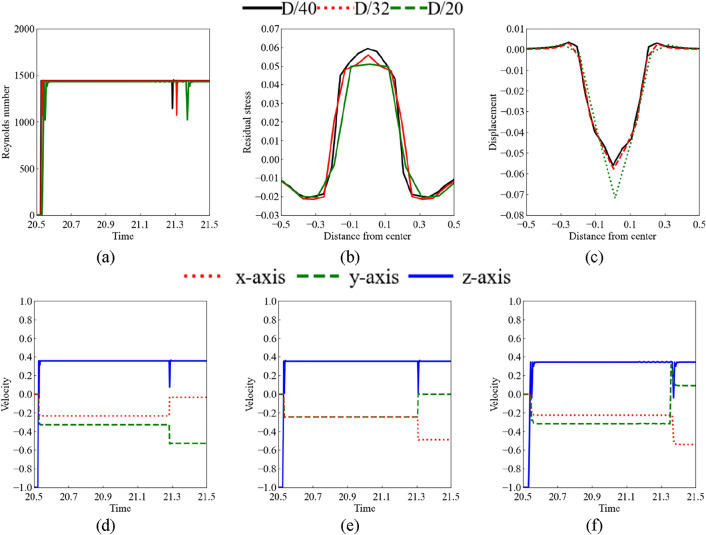
The influence of computational grid size on the random motion of the shot after the impact: (**a**) relative Reynolds number, (**b**) residual stress, (**c**) indentation profile, and non-dimensional time history of normalized shot velocity: (**d**) D/40, (**e**) D/32, and (**f**) D/20.

## Data Availability

The datasets used and/or analyzed during the current study available from the corresponding author on reasonable request.

## Appendix 1

In addition to the baseline grid size D/40 used in the main text, we considered coarser grid resolutions of D/32 and D/20. Figure 14a shows a time history of the relative Reynolds number. It was confirmed that the random motion of the shot after the impact occurs regardless of the grid resolution, although the magnitude and direction of the motion are different. In addition, the asymmetric distribution of the structure surface was also reproduced for D/32 and D/20. As shown in Fig. 14b and c, the higher the crater rim, the earlier the random motion of the shot begins. Figure 14d–f show a time history of the shot velocity after the shot impacts. The time at which the random motion of the shot occurs is influenced by the shape of the impact crater edge. In summary, the random motion of the shot is physically consistent; however, its magnitude and timing of occurrence depend on numerical factors such as grid resolution.
